# The outcome of kyphosis tuberculosis treated with one stage reconstruction surgery. A case series

**DOI:** 10.1016/j.ijscr.2019.07.053

**Published:** 2019-07-26

**Authors:** Fachrisal, Luthfi Gatam, Asrafi Rizki Gatam, Aji Antoro

**Affiliations:** Department of Orthopaedic and Traumatology, Fatmawati Hospital, Jakarta, Indonesia

**Keywords:** Modified lateral extracavitary approach, Kyphotic deformity, Anterior column reconstruction

## Abstract

**Introduction:**

Commonly, two stage combined anterior - posterior approaches were performed to treat significant kyphotic deformity, but potentially increase morbidity level. Recently, single - stage posterior approach for anterior column reconstruction have shown sagittal alignment improvement. The objective of this study is to describe a series of kyphotic deformity patients whom were treated using modified lateral extracavitary approach for anterior column reconstruction and posterior instrumentation.

**Methods:**

Data collected from all kyphotic deformity patients whom treated with modified lateral extracavitary approach between 2016 until 2017 and this research work has been reported in line with the PROCESS criteria. In addition this technique could address kyphotic correction.

**Results:**

7 patients were reported, 4 males and 3 females with local and regional kyphotic more than 40 degrees. Procedures performed mostly on the right side to avoid the aorta with approximately 2 cm of the rib distal to the transverse processes. The average estimated blood loss and length of surgery were 1280 ml and 3.9 h. None of the patient had neurological deficit, and all of them have shown kyphotic improvement.

**Conclusion:**

The modified lateral extracavitary approach for anterior column reconstruction and posterior instrumentation is a viable alternative to the standard combined approach. This approach continues to evolve as instrumentation development and possesses significant advantages.

## Highlights

• Kyphotic deformity is common and can be associated with considerable morbidity.
• Vertebral resection and reconstruction have been shown to preserve neurological function and decrease pain.
• Two-stage, combined anterior and posterior approaches are performed to surgically address significant vertebral kyphotic.
• The modified lateral extracavitary approach for reconstruction and instrumentation is an alternative to standard approach.

## 1 Introduction

Kyphotic deformity is common and can be associated with considerable morbidity, this deformity usually caused by spinal tuberculosis in most parts of the world [1]. Although chemotherapy is highly effective in controlling tubercular infection, patients treated with chemotherapy alone have an average increase of 15° in deformity and 3%–5% of the patients develop kyphosis greater than 60° [2]. A severe kyphosis can lead to immense cosmetic and psychological disturbance in growing children and can result in costo-pelvic impingement, secondary cardio-respiratory problems and late-onset paraplegia. Correction of an established kyphosis is both difficult and hazardous with a high rate of complications, even in experienced hands. It is essential that prevention of deformity be an integral part of any treatment schedule in spinal tuberculosis [3,4].

Corrective surgery can be done only in patients in whom the deformity was severe, active disease still present, paraplegia or death from chest complications imminent. Vertebral resection and reconstruction have been shown to preserve neurological function and decrease pain [5,6]. Most commonly, two-stage, combined anterior and posterior approaches are performed to surgically address significant vertebral kyphotic [7,8].

Later refinement of surgical techniques, development of newer approaches and availability of rigid spinal instrumentation made single stage correction of established deformity relatively safe and a procedure with good outcome. Transpedicular decancellation osteotomy, Pedicle subtraction osteotomy, Direct internal kyphectomy have been used to treat kyphosis in active as well as healed disease [9,10]. Anil Jain et al. reported kyphosis correction through an extra-pleural, antero-lateral (costo-transversectomy) approach with mean kyphosis correction of 27.3° and no persistent neurodeficits. Bezer et al. used a Transpedicular decancellation osteotomy to correct post tuberculous kyphotic with no neural complications. The objective of this study is to describe a series of kyphotic deformity patients whom were treated using single stage correction using modified lateral extracavitary approach for anterior column reconstruction and posterior instrumentation [1,11,12].

## 2 Methods

We collected all patients with kyphotic deformity who were treated with modified extracavitary approach for anterior column vertebra reconstruction using cage and posterior instrumentation in between 2016 until 2017 and this research work has been reported in line with the PROCESS criteria [13]. The diagnosis of kyphotic deformity was made based on X-ray and MRI with local and regional kyphotic perimeters. Post operative kyphotic correction were measured by X-ray examination after surgery which the normal kyphotic degree in thoracal curve was 10^0^–40^0^. In other hand we also collected demographic data of patients, type of the disease, intraoperative blood loss, length of operation time, neurological status, length of stay patient, cost of operation.

The techniques of double fixation for posterior instrumentation and anterior column reconstruction using lateral extracavity approach in our hospital was performed with the following procedures:

• Laminectomies are performed at the level of the anterior column resection.
• Surrounding facets are removed to expose the disc spaces and the transverse processes, a small segment of the ribs, and rib heads are resected on the side of the approach at the level of the resection.
• Discectomy, and corpectomy was performed to resect the anterior column of vertebrae
• Pedicle screw fixation was inserted in the 2–3 level vertebrae at upper and lower resection level.
• The kyphotic correction was made by pedicle rod system after the segment resection was mobile.
• The anterior column reconstruction was performed with cage instrumentation which inserted from right side resection area in between the nerve roots.
• The pedicle screw over the resection area was compressed to traps the cage fixation.
• In the end, the C-arm examination was performed to confirm the positional fixation of cage and pedicle screw.

## 3 Results and discussion

The study included 7 patients with kyphotic deformity. They consist of 4 males and 3 females with the mean age 26.2 years old (range 14–29 years old). 6 patients with spondylitis tuberculosis, and 1 patient with metastasis bone disease. The lesion location of 6 cases at the thoracal vertebra, and 1 case at the thoracolumbal vertebra. The mean of local/regional kyphotic pre operation was 45.2^0^ (range 42–53^0^). From neurological status, 4 patients with Frankel B, 2 patients with Frankel C, and 1 patient with Frankel D. The mean estimated blood loss was 1280cc (range 1100cc–1700cc). The mean length of surgery was 3.9 h (range 3.7–4.5 h). The mean length of stay in the hospital was 4.5 days (range 4–7 days). None of the patient had neurological deficit, and all of them have shown kyphotic improvement with mean local/regional kyphotic post operation was 30.5^0^ (range 28°–35°).

**Fig. 1:**
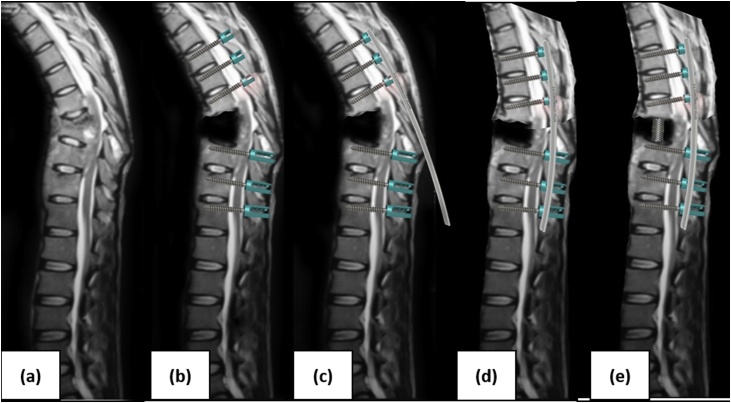
Anterior column reconstruction and posterior instrumentation illustration (lesion and kyphotic at thoracal 8–9 vertebra). (a) Resection at lesion level thoracal 8–9 vertebra. (b) Pedicle screw placement at 3 level vertebra upper and lower from resection area. (c) Inserting rod to the pedicle screw fixation. (d) Kyphotic correction with pedicle rod system. (e) inserting cage to resection area to done the anterior reconstruction.

The posterior approach to the anterior spine has long been an attractive option for spinal surgeons (Fig. 1). The modified lateral extracavitary approach was developed in part by Norman Capener and then modified by Sanford Larson and others. Many of its advantages arise from the ability to avoid morbidity associated with anterior or lateral incisions. This is particularly important in the oncologic and infecton setting because many of the patients have already had interventions such as surgery, chemotherapy, or radiation that can compromise pulmonary function or increase the difficulty of gaining exposure. Though versatile, the lateral extracavitary approach is technically challenging, associated with high blood loss and wound-healing problems, and anterior cage placement is often smaller than that in traditional anterior exposure [1,10,11].

**Fig. 2:**
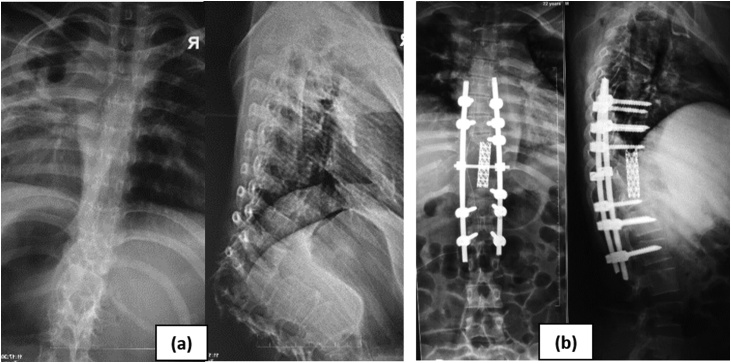
Kyphotic correction with modified lateral extracavitary approach. (a) Preoperative Xray-MRI with local/regional kyphotic 90.30, and lesion at Th11-L1 vertebrae due to spondylitis tuberculosis. (b) Post operative Xray with correction local/regional kyphotic 26.50.

In our series, we successfully used the lateral extracavitary approach to perform circumferential anterior column reconstruction for kyphotic deformity, with an anterior cage and supplemental instrumented posterior at least two - three levels above and below resection (Fig. 2). The average blood loss was 1280 ml. This is better compare to the 1580 ml mean EBL reported by Xu et al. who used transpedicular or lateral extracavitary approaches. It is also in the range of values reported by Lu et al. (1320 ml), and Wang et al. (1250 ml). Our technique was different than the classical lateral extracavitary approach performed through a paramedian incision. The incision and subsequent maneuvers that we employed are similar to those described by Snell et al. It was critical that we used a midline incision: this allowed us to work without significant impedance from the scapula. Furthermore, the midline approach facilitated a circumferential access to resection [12,14,15].

## 4 Conclusion

The modified lateral extracavitary approach for anterior column reconstruction and posterior instrumentation is a viable alternative to the standard combined approach. This approach continues to evolve as instrumentation development and possesses significant advantages. Further study need to be conducted with more sample sizes, and long time period to observed the fixation endurance and clinical outcome of patients.

## Funding

There is no sources of funding sponsor in this manuscript.

## Ethical approval

The authors have no ethical conflicts to disclose.

## Consent

Written informed consent was obtained from the patients for publication of this case report and accompanying images. A copy of the written consent is available for review by the Editor-in-Chief of this journal on request.

## Author contribution

1. Fachrisal, MD. Contributed as making the conceptualization, data curation, study design, funding acquisition, supervision, and final approval of manuscript.

2. Luthfi Gatam, MD. Contributed as making the conceptualization, data curation, study design, funding acquisition, supervision, and final approval of manuscript.

3. Asrafi Rizki Gatam, MD. Contributed as making the study design, collecting, investigationing, and analyzing the data, formal analysis, writing manuscript.

4. Ajiantoro, MD. Contributed as making the study design, collecting, investigationing, and analyzing the data, formal analysis, writing manuscript.

## Registration of research studies

researchregistry5015, and you will find your registration here: https://www.researchregistry.com/browse-the-registry#home/ Please store this email for reference.

## Guarantor

Fachrisal, MD.

Luthfi Gatam, MD.

## Availability of data and materials

All data generated or analyzed during this study are included in this published article.

## Declaration of Competing Interest

The authors have no ethical conflicts to disclose.
